# Thanks to all those who reviewed for Journal of Medical Case Reports in 2014

**DOI:** 10.1186/s13256-015-0531-x

**Published:** 2015-02-12

**Authors:** Michael R Kidd

**Affiliations:** Faculty of Health Sciences, Flinders University, GPO Box 2100, Adelaide, SA 5001 Australia

## Abstract

A peer-reviewed journal would not survive without the generous time and insightful comments of the reviewers, whose efforts often go unrecognized. Although final decisions are always editorial, they are greatly facilitated by the deeper technical knowledge, scientific insights, understanding of social consequences, and passion that reviewers bring to our deliberations. For these reasons, the Editor-in-Chief and staff of the journal warmly thank the 906 reviewers whose comments helped to shape *Journal of Medical Case Reports*, for their invaluable assistance with review of manuscripts for the journal in Volume 8 (2014).

**Souheil Abdel Nour**

USA

**Ahmad Abdelkarim**

USA

**Rohana Abdul Ghani**

Malaysia

**Koji Abe**

Japan

**Arnis Abolins**

Latvia

**Edward Abraham**

USA

**Yushi Adachi**

Japan

**Kensuke Adachi**

Japan

**Roberto Adani**

Italy

**Omotayo Adesiyun**

Nigeria

**Christian Agard**

France

**Elugwuraonu Agbakwuru**

Nigeria

**Varun Agrawal**

USA

**Tasnim Ahsan**

Pakistan

**Toshimi Aizawa**

Japan

**Sinem Akgül**

Turkey

**Esen Akpek**

USA

**Gabriel Alcala**

Colombia

**Mohammad Al-Haggar**

Egypt

**Waleed Alkhudair**

Saudi Arabia

**Fawzia Al-Otaibi**

Saudi Arabia

**Murat Alper**

Turkey

**Mohannad Alqudah**

Jordan

**Fatih Altunrende**

Turkey

**Hernando Raphael Alvis Miranda**

Colombia

**Theodore Anagnostou**

Greece

**Olympia Anastasiou**

Germany

**Uzel Andre-Pierre**

Guadeloupe

**Manjula Annappa**

UK

**Alexandros Apostolopoulos**

Greece

**Slobodan Apostolski**

Serbia

**Michele Arcopinto**

Italy

**Sandro Ardizzone**

Italy

**Guillaume Armengol**

France

**Eleni Arnaoutoglou**

Greece

**Vaithi Arumugaswami**

USA

**Keyhan Ashrafi**

Iran

**Irene Asouhidou**

Greece

**Jaromir Astl**

Czech Republic

**Andrew Atkinson**

USA

**Stephen Attard**

Malta

**Brian Austin**

UK

**Mehmet Aydogan**

Turkey

**Hüseyin Ayhan**

Turkey

**Yavuz Ayhan**

Turkey

**Ibrahim Azboy**

Turkey

**Hideki Azuma**

Japan

**Hisatoshi Baba**

Japan

**Yuh Baba**

Japan

**Hector Baillie**

Canada

**Salar Bakhtiyari**

Iran

**Khaled Balam**

Egypt

**Alberto Baldini**

Italy

**Nikolaos Baltayiannis**

Greece

**Upali Banagala**

Sri Lanka

**Paul Anthony Banaszkiewicz**

UK

**Paulina J.M. Bank**

Netherlands

**Giorgio Barbarini**

Italy

**Mary Barry**

Ireland

**Sandip Basu**

India

**Mihaela Batke**

USA

**Mark Bechtel**

USA

**George Beiko**

Canada

**Diana Bell**

USA

**Reuben Benjamin**

UK

**Ronald Benveniste**

USA

**Alan Berger**

Canada

**Thomas Berger**

Switzerland

**Robert Bergholz**

Germany

**Achim Berthele**

Germany

**Rajesh Bhagat**

USA

**Vish Bhattacharya**

UK

**Shahaab Bhatti**

UK

**Deepak Bhonagiri**

Australia

**Can Bicmen**

Turkey

**Jovan Bila**

Serbia

**Ashish Bindra**

India

**Jean Bismuth**

USA

**Benoit Blondeau**

USA

**Stijn Blot**

Belgium

**Paolo Boffano**

Italy

**Massimo Bonacchi**

Italy

**Hamouda Boussen**

Tunisia

**David Boyer**

USA

**Colin Bradley**

Ireland

**Patrick Bradley**

UK

**Elizabeth Brant**

USA

**Milan Brazdil**

Czech Republic

**Massimo Breccia**

Italy

**Olivier Brehant**

France

**Raoul Breitkreutz**

Germany

**Vesna Breznik**

Slovenia

**Giuseppe Brisinda**

Italy

**Konrad Brockmeier**

Germany

**Claudia Bruè**

Italy

**Manjunatha Bs**

India

**Alicja Buczek**

Poland

**Soetjiningsih Budiarsa**

Indonesia

**Ertan Bulbuloglu**

Turkey

**Orhan Bulut**

Denmark

**Jean Burr**

USA

**Cen Bytyqi**

Albania

**Maurizio Calcagni**

Switzerland

**Massimo Caldarelli**

Italy

**Mario Camarda**

Italy

**Danny Camfferman**

Australia

**Giovanni Cammarota**

Italy

**Fabio Cesare Campanile**

Italy

**Alessandro Cannavale**

Italy

**Steve Carlan**

USA

**Pilar Carranza**

Mexico

**Santos Castañeda**

Spain

**Joaquin Castro**

Spain

**Juan Cataño**

Colombia

**Niyazi Cebi**

Germany

**Francesco Ceccarelli**

Italy

**Marco Cei**

Italy

**Giuliano Cerulli**

Italy

**Camila Cespedes**

Colombia

**Parin Chaivisuthangkura**

Thailand

**Shanmuganathan Chandrakasan**

USA

**Shi-Min Chang**

China

**Philippe Chanson**

France

**Rene Chapot**

Germany

**Alexandros Charalabopoulos**

UK

**Keerati Chareancholvanich**

Thailand

**Stylianos Chatzipanagiotou**

Greece

**Nikolaos Chavouzis**

Greece

**Baoan Chen**

China

**Ying-Sheue Chen**

Taiwan

**Domenico Chirchiglia**

Italy

**Joanne Chiu**

Hong Kong

**Kyu-Sup Cho**

South Korea

**Anand Chockalingam**

USA

**Evangelos Cholongitas**

Greece

**Tumul Chowdhury**

Canada

**Sara Ciccone**

Italy

**Mislav Cimic**

Croatia

**Kristl Claeys**

Germany

**Christelle Clement-Duchene**

France

**Joannes Clerinx**

Belgium

**David Cloke**

UK

**Paul Cockwell**

UK

**Stephan Cohen-Bacrie**

France

**Demosthenes Cokkinos**

Greece

**Andrés Combalia**

Spain

**Stefano Congiu**

UK

**Patricia Conville**

USA

**Ruggero Corso**

Italy

**Demet Coskun**

Turkey

**Ana Costa**

Brazil

**Anna Crocco**

Italy

**Adam Croft**

UK

**Kimberly Crowder**

USA

**Salvador Cruz-Flores**

USA

**Francisco Cuellar-Ambrosi**

Colombia

**Vatsla Dadhwal**

India

**Antonio D’alessandro**

Italy

**Maurizio D’amario**

Italy

**Abdul Qayoom Dar**

India

**Giuseppe Dattilo**

Italy

**Carlo De Angelis**

Canada

**Emmanuel De Laere**

Belgium

**Francesco Giuseppe De Rosa**

Italy

**Dulantha De Silva**

Sri Lanka

**Juan Carlos De Vicente**

Spain

**Masataka Deie**

UK

**Andreas K. Demetriades**

UK

**Ismail Demiryilmaz**

Turkey

**Francesco Dentali**

Italy

**Aparna Deshpande**

India

**Giovambattista Desideri**

Italy

**Mark Detweiler**

USA

**Amar Dhand**

USA

**Luca Di Antonio**

Italy

**Andrea Di Bernardo**

Italy

**Hendrik Dienemann**

Germany

**Daan Dierickx**

Belgium

**Panagiotis Dikeakos**

Greece

**Ludovico Docimo**

Italy

**Stefanos Dokas**

Greece

**Bessy Domi**

Greece

**Frank Domino**

USA

**William Donaldson**

UK

**Kieran Doran**

Ireland

**Dimitrios Dougenis**

Greece

**Panagiotis Drakopoulos**

Switzerland

**Georgios Drosos**

Greece

**Charalambos Drossos**

Greece

**Balazs Duga**

Hungary

**Gregory Dumanian**

USA

**Reinhard Dummer**

Switzerland

**Martin Dunser**

Austria

**Nizam Duran**

Turkey

**Onur Burak Dursun**

Turkey

**Amaka Edeani**

USA

**Suzanne Edmunds**

USA

**Dr Meera Ekka**

India

**Fatih Eksioglu**

Turkey

**Ghada El Sagheer**

Saudi Arabia

**Aravindan Elangovan**

India

**Abdelhamid Elgazzar**

Kuwait

**Adel El-Naggar**

USA

**Mohamed Emad**

Egypt

**Tze Kiong Er**

Taiwan

**Silvano Esposito**

Italy

**Giuseppe Maria Ettorre**

Italy

**Mohamed Fahmy**

Egypt

**Lee Fairlie**

South Africa

**Rafik Fanous**

UK

**Silvia Fargion**

Italy

**Eriberto Farinella**

Italy

**James Farmer**

Canada

**Magi Farre**

Spain

**Shoaib Faruqi**

UK

**Hasan Fattah**

USA

**Chris Fearne**

Malta

**Pasquale Ferrante**

Jamaica

**Mara Ferrara**

Italy

**Mauricio Ferri**

Canada

**Nicholas Fischer**

New Zealand

**Horace Fletcher**

Jamaica

**Panagiota Flevari**

Greece

**Andrés Fonnegra**

Colombia

**Denis Leonardo Fontes Jardim**

Brazil

**Alessia Fornoni**

USA

**Elias Fotiadis**

Greece

**Christina Fotopoulou**

UK

**Claudio Fozza**

Italy

**Gabriele Fragasso**

Italy

**Dario Frank**

Germany

**Florian Friedmacher**

Ireland

**Johan H.M. Frijns**

Netherlands

**Lorenzo Fuccio**

Italy

**Masashi Fukushima**

Japan

**Ioannis Galanis**

Greece

**Paolo Gallipoli**

UK

**Sean Galvin**

Australia

**T. Clark Gamblin**

USA

**Prasanth Ganesan**

India

**Musa Abubakar Garbati**

Saudi Arabia

**Rakesh Garg**

India

**Enrique Garrido**

UK

**Bogdan Gaspar**

Romania

**Amy Gassama Sow**

Senegal

**Danielle Gatti**

USA

**Elzbieta Gawrych**

Poland

**Roman Gebauer**

Germany

**Ralph George**

Canada

**Petros Georgopoulos**

Greece

**Mehdi Ghasemi**

USA

**Mohammad Ghasemi-Rad**

Iran

**Christopher Ghazala**

UK

**Jamileh Ghoddusi**

Iran

**Kemel Ahmed Ghotme Ghotme**

Colombia

**Stilianos Giannakopoulos**

Greece

**Paul Gibson**

Canada

**Don Gilden**

USA

**Ritu Gill**

USA

**Matteo Giorgi**

Italy

**Olga Giouleme**

Greece

**Stefano Giulieri**

Switzerland

**Irina Giurgea**

France

**Panagiotis Givissis**

Greece

**Markus Gödel**

Germany

**Bee See Goh**

Malaysia

**Cem Gökçen**

Turkey

**Elizabeth Golesic**

Canada

**Rodrigo Gomes Da Silva**

Brazil

**John Gonder**

Canada

**Laura González-García**

Spain

**Ponnampalam Gopalakrishnakone**

Singapore

**David Gorard**

UK

**Martijn Pieter Gosselink**

Netherlands

**Pierre Goussard**

South Africa

**Tarang Goyal**

India

**Elvira Grandone**

Italy

**Davide Grassi**

Italy

**Victor Grech**

Malta

**Neil Greening**

UK

**Sandeep Grover**

USA

**Beatriz Guglieri**

Spain

**Farshid Guilak**

USA

**Dorothy Gujral**

UK

**Niti Gulati**

India

**Selma Guler**

Turkey

**Anju Gupta**

India

**Gaurav Gupta**

USA

**Manish Gupta**

India

**Swati Gupta Bhattacharya**

India

**Kathleen Gura**

USA

**José-María Gutiérrez**

Costa Rica

**Melih Güven**

Turkey

**Vijay Hadda**

India

**Hind Hadi**

Iraq

**Sanghoon Han**

South Korea

**Tharangrut Hanprasertpong**

Thailand

**Rehan Haq**

India

**K.V.S. Hari Kumar**

India

**Uma Hariharan**

India

**Claire Harrison**

UK

**Yamada Harumoto**

Japan

**Junichi Hashimoto**

Japan

**Miroslav Haspl**

Croatia

**Iffat Hassan**

India

**Michael Hawkes**

Canada

**Reda Hemida**

Egypt

**Patrick Henn**

Ireland

**Ruth Heying**

Belgium

**Catherine Hill**

Australia

**Andreas Himmelmann**

Switzerland

**Simon Hjerrild**

Denmark

**Martin Hoenigl**

Austria

**Iva Hojak**

Croatia

**Sung-Tae Hong**

South Korea

**Jae Young Hong**

South Korea

**Hironobu Hoshino**

Japan

**Sion Houri**

Israel

**Sun Huh**

South Korea

**Robert Hunter**

UK

**Mark Huntington**

USA

**Richard Huntsman**

Canada

**Nexhmi Hyseni**

Kosovo

**Giorgio Iannetti**

Italy

**Kenneth Iczkowski**

USA

**Abdullah Inal**

Turkey

**Philip Ind**

UK

**Yasushi Innami**

Japan

**Sebastian Ionescu**

Romania

**Fumihiro Ishida**

Japan

**Jafar Jafari**

UK

**Amita Jain**

India

**Neetu Jain**

India

**Praveen Jajoria**

USA

**Jens Jakob**

Germany

**Prashant Jani**

Canada

**Anis Jellad**

Tunisia

**Seong Woo Jeon**

South Korea

**Woo-Jin Jeong**

South Korea

**Verónica Jeraldo**

Brazil

**Andrei Joaquim**

Brazil

**Ken Johkura**

Japan

**Brian Joondeph**

USA

**Tatjana Josifova**

Switzerland

**Eui Dal Jung**

South Korea

**Fevziye Kabukcuoglu**

Turkey

**Anish P. Kadakia**

UK

**Abdullah Kaki**

Saudi Arabia

**Arjun Kakrani**

India

**Evagelos Kalfarentzos**

Greece

**Michal Kaliszan**

Poland

**Takeharu Kanazawa**

Japan

**Gokul Vignesh Kanda Swamy**

UK

**Hideaki Kanemura**

Japan

**Yoshihiko Kanno**

Japan

**Amrinderjit Kanwar**

India

**Rene Kanza**

Canada

**Emmanouil Ioannis Kapetanakis**

Greece

**Pinar Karabagli**

USA

**Nesrin Karabul**

Germany

**Panagiotis Karanis**

Germany

**Mehdi Karkouri**

Morocco

**Maria Karouki**

Greece

**Suneth Karunarathne**

Sri Lanka

**Imad Kassis**

Israel

**Masaya Kawai**

Japan

**Leticia Kawano-Dourado**

Brazil

**Cemil Kayali**

Turkey

**Carolin Kayser**

Germany

**Donald Kearns**

USA

**Jean Keddissi**

USA

**Pierre Kehr**

France

**Martina Kelly**

Canada

**Michael Kelly**

USA

**Ozgur Kemik**

Turkey

**Melita Kenealy**

Australia

**Mahmoud Khattab**

Egypt

**S. Guan Khoo**

Ireland

**Teruyo Kida**

Japan

**Katarzyna Kilis-Pstrusinska**

Poland

**Tae-Heung Kim**

South Korea

**Hee Jun Kim**

South Korea

**Won-Jang Kim**

South Korea

**Seong Heon Kim**

South Korea

**Ryo Kimura**

Japan

**Ana Cristina King Martinez**

Mexico

**Takeshi Kinjo**

Japan

**Laszlo Kiraly**

United Arab Emirates

**Boris Kirshtein**

Israel

**Kamal Kishore**

USA

**Hiroaki Kitagawa**

Japan

**Masaru Kitamura**

Japan

**Ingo Kleiter**

Germany

**Richard Knight**

UK

**Christian Koch**

USA

**Puneet Kochhar**

India

**Diamantis Kofteridis**

Greece

**Anna Kohn**

Italy

**Mehmet Hanifi Kokaçya**

Turkey

**Matthias König**

Switzerland

**Satoshi Kono**

Japan

**Marina Kontogiorgi**

Greece

**Marcela Kopacova**

Czech Republic

**Ioannis Kornezos**

Greece

**Chris Kosmidis**

UK

**Prakash Kotagal**

USA

**Anastasia Kotanidou**

Greece

**Efstathios Kotidis**

Greece

**Kalliopi Kotsa**

Greece

**Katerina Kotzampassi**

Greece

**Georgios Kouliatsis**

Greece

**Gregory Kouraklis**

Greece

**Ioannis Koutelidakis**

Greece

**Euphrosyni Koutsouraki**

Greece

**Anastasios Koutsovasilis**

Greece

**Gopal Kowdley**

USA

**Roland Kreis**

Switzerland

**Uma Krishnamoorthy**

UK

**David Krpata**

USA

**Ikuko Kubokawa**

Japan

**Senanayake Kularatna**

Sri Lanka

**Ajay Kumar**

India

**Mahesh Kumar**

UK

**Niranjan Kumar**

Kuwait

**Shivaani Kummar**

USA

**Motoki Kuramochi**

Japan

**Mertihan Kurdoglu**

Turkey

**Vivek Kute**

India

**Yok-Lam Kwong**

Hong Kong

**Severin Laeuchli**

Switzerland

**De-Hua Lai**

China

**Ka On Lam**

Hong Kong

**William Laskin**

USA

**Ernest Lau**

UK

**Andreas Lazaris**

Greece

**Laurianne Le Gloan**

France

**David Lebeaux**

France

**Jaekwang Lee**

South Korea

**Claudio Letizia**

Italy

**Yuen-Fai Leung**

Hong Kong

**Roland Leung**

Hong Kong

**Liran Levin**

Israel

**Miles Levy**

UK

**Chengyao Li**

China

**Wei Li**

China

**Xiaoling Liang**

China

**Ying Liang**

China

**Christos Liapis**

Greece

**Chester Lie**

Hong Kong

**Sue Lightman**

UK

**Lassi Liikkanen**

Finland

**Jin-Ching Lin**

Taiwan

**James Lin**

USA

**Tung-Liang Lin**

Taiwan

**Gopal Lingam**

Singapore

**Katerina Linhartova**

Czech Republic

**Qun Liu**

China

**Bingqian Liu**

China

**Tianrun Liu**

China

**Eddie Liu**

Canada

**Quan Liu**

China

**Tsafrir Loebl**

Israel

**Jonathan Loughead**

UK

**Chih-Ming Lu**

Taiwan

**Shue-Fen Luo**

Taiwan

**Stefan Lüth**

Germany

**Ângela Cristina Malheiros Luzo**

Brazil

**Hatsuhiko Maeda**

Japan

**Yoshito Maeno**

Japan

**John Maesaka**

USA

**Rama Maganti**

USA

**Magboul Magboul**

USA

**Bridget Maher**

Ireland

**Joseph Maher**

USA

**Kohei Makita**

UK

**Demosthenes Makris**

France

**Nilesh Makwana**

UK

**Vijay Malpathak**

India

**Efstratios Maltezos**

Greece

**Vincenzo Dario Mandato**

Italy

**Charles Mango**

USA

**Nicholas Mann**

UK

**Surendra Mantoo**

Singapore

**Emilia Manzato**

Italy

**Giorgos Margaritopoulos**

Greece

**Athanasios Marinis**

Greece

**Gian Luigi Marseglia**

Italy

**Ian Marshall**

Australia

**Thomas Martin**

UK

**Hirofumi Maruyama**

Japan

**Helena Marzo-Ortega**

UK

**Lorenzo Masi**

Italy

**Ibrahim Masoodi**

Saudi Arabia

**Sotiria Mastoraki**

Greece

**Nilesh Mathuria**

USA

**Ratko Matijevic**

Croatia

**Andreas Mavrogenis**

Greece

**Michinori Mayama**

Japan

**Joni Mazza-Mccrann**

USA

**Maria Antonietta Mazzei**

Italy

**Valeria Mazzi**

Italy

**Sam Mbulaiteye**

USA

**Meer Chisthi Meeran Pillai**

India

**Bruno Megarbane**

France

**Enawgaw Mehari**

USA

**Changlin Mei**

China

**Jurka Meichtry**

Switzerland

**Shimon Meretyk**

Israel

**Andrew Merkur**

USA

**Linda Metaxa**

Greece

**Salvatore Micali**

Italy

**Othon Michail**

Greece

**Jaroslav Michalek**

Czech Republic

**Pavel Michalek**

Czech Republic

**Pavel Michalek**

UK

**Antonios Michalopoulos**

Greece

**Nickolaos Michalopoulos**

Greece

**Giancarlo Micheletto**

Italy

**Atsuya Miki**

Japan

**Ioannis Mikoniatis**

Greece

**Stephanos Milias**

Greece

**Michael Mimouni**

Israel

**Akihito Minamide**

Japan

**Roberta Minelli**

Italy

**Alberto Minetti**

Italy

**Michele Miraglia Del Giudice**

Italy

**George Mistriotis**

Greece

**Jayanta Mitra**

India

**Amit Mittal**

India

**Seiki Miura**

Japan

**Hiroshi Miyamoto**

USA

**Muhajir Mohamed**

Australia

**Mohamed Ali Ahmed Mohamed**

Egypt

**Rhajaoui Mohamed**

Morocco

**Khalid Mohammad**

USA

**Ali Mokhtarpour**

Malaysia

**Felix Momm**

Germany

**Suneeta Monga**

Canada

**Guillermo Monsalve**

Colombia

**Benvon Moran**

Canada

**Carthage Moran**

Ireland

**Mohib Morcos**

Canada

**Tom Moreels**

Belgium

**Umberto Morelli**

France

**Arsim Morina**

Kosovo

**Hideo Morioka**

Japan

**Silvia Morlino**

Italy

**Eleni Mouloudi**

Greece

**Ramanuj Mukherjee**

India

**Nicola Mumoli**

Italy

**James Munthali**

Zambia

**Giuseppe Murdaca**

Italy

**Thibaut Murez**

France

**Heather Murphy-Lavoie**

USA

**Nagarajan Muthialu**

UK

**Seema Nagpal**

USA

**Akitoshi Nakashima**

Japan

**Raj Nandi**

UK

**Sameer Naranje**

USA

**Kiran Narreddy**

USA

**Asma Nasim**

Pakistan

**Mc Nassogne**

Belgium

**Sundaram Natarajan**

India

**Ramon Navarro**

United Arab Emirates

**Andrew Neviaser**

USA

**Jarungchit Ngamphaiboon**

Thailand

**Korahanis Nicolas**

France

**Lluís Nisa**

Switzerland

**Aamer Nisar**

UK

**Tetsuro Nishimura**

Japan

**Pavel Nockel**

USA

**Ahmad Nofal**

Egypt

**Hilali Noordeen**

UK

**Aqodad Nourdin**

Morocco

**Soliman Noureldin**

UK

**Nader Nouri-Majalan**

Iran

**Muhammed Mubarak Nuhari**

Pakistan

**Brian O’Donnell**

Ireland

**Olusola Oduntan**

USA

**Kayode Oduwole**

Ireland

**Yutaka Ogata**

Japan

**Masafumi Ohki**

Japan

**Ridvan Oksayan**

Turkey

**Francesco Oliva**

Italy

**Daniel Olmedo**

Argentina

**Kathleen Olson**

USA

**Sukru Sadik Oner**

Turkey

**Carlos Eduardo Ontiveros**

Brazil

**Anil Thomas Oommen**

India

**Ambrogio Orlando**

Italy

**Norberto Ortego-Centeno**

Spain

**Melissa Osborn**

USA

**Zdenko Ostojic**

Bosnia and Herzegovina

**Takanobu Otsuka**

Japan

**Toshinori Ozaki**

Japan

**Aynur Özge**

Turkey

**David Pace**

Malta

**Clifford Packer**

USA

**Ivan Padjen**

Croatia

**Dasja Pajkrt**

Netherlands

**Ajay Pal Singh**

India

**Aparna Palit**

India

**Jose Prisco Palma-Nicolas**

Mexico

**Periklis Panagopoulos**

Greece

**Vijay Panchanadikar**

India

**Padmakar Pandit**

India

**Amit Pandya**

USA

**Stavros Panidis**

Greece

**Maria Panopoulou**

Greece

**Vasileios Papadopoulos**

Greece

**Kostas Papagiannopoulos**

UK

**Theodossis S. Papavramidis**

Greece

**Basilios Papaziogas**

Greece

**Spyros Papiris**

Greece

**Dimitrios Paraskevopoulos**

UK

**Paolo Paredi**

UK

**Lawrence Parish**

USA

**Jeong-Yeol Park**

South Korea

**Jung Gil Park**

South Korea

**Jong Sung Park**

South Korea

**Martyn Parker**

UK

**Rocio Parody**

Spain

**Nigel John Parr**

UK

**Marco Pasini**

Italy

**Dave Paskar**

Canada

**Dritan Pasku**

Greece

**Gregorios Paspatis**

Greece

**Salvatore Patanè**

Italy

**Anil Pathare**

Oman

**Anil Patki**

India

**Friedemann Paul**

Germany

**Catherine Pennington**

UK

**Nadia Peparini**

Italy

**Bruno Pereira**

Brazil

**Ajantha Perera**

Sri Lanka

**Nilanka Perera**

Sri Lanka

**Maria Asuncion Perez-Jacoiste**

Spain

**Aleksandar Peric**

Serbia

**Christian-Dominik Peterlein**

Germany

**Adriano Piattelli**

Italy

**Gabriele Piffaretti**

Italy

**Promod Pillai**

USA

**Paolo Pillastrini**

Italy

**Trinitario Pina Murcia**

Spain

**Antonio Pinto**

Italy

**Chrysoula Pipili**

Greece

**Francesco Pisani**

Italy

**Mauro Pittiruti**

Italy

**Benjamin Piura**

Israel

**Domenico Plantone**

Italy

**Mike Polyzonis**

Greece

**A. Ponnuswamy**

UK

**Vera Popovic**

Serbia

**Ramin Pourghorban**

Iran

**Harry Powell**

UK

**Juan Carlos Prados-Frutos**

Spain

**Manousos-Georgios Pramateftakis**

Greece

**Sampath Chandra Prasad**

India

**Carlo Pratesi**

Italy

**Karl-Josef Prommersberger**

Germany

**Anna Punga**

Sweden

**Sohail Qayyum**

USA

**Aldo Quattrone**

Italy

**Ahmed Rabie**

Egypt

**Jasim Radhi**

Canada

**Annie Rajaratnam**

India

**Juan Fernando Ramon**

Colombia

**Serramito Ramon**

Spain

**Bruce Ramshaw**

USA

**Murali Rangaswami, Tanjore**

India

**Piyush Ranjan**

India

**Anirudh Rao**

UK

**Amrith Rao**

UK

**Dimitrios Raptis**

Germany

**Serge Rasskazoff**

USA

**Barbara Rath**

Germany

**Chamara Ratnayake**

Sri Lanka

**Hilpi Rautelin**

Sweden

**Richter Razafindratsimandresy**

Madagascar

**Massimo Re**

Italy

**Krishna Reddy**

UK

**Nishitha Reddy**

USA

**Neil Reeves**

UK

**Roberto Ria**

Italy

**Benjamin Ricciardi**

USA

**Jonathan Riess**

USA

**Richard Rison**

USA

**Hisashi Ro**

Japan

**Simon Benedict Roberts**

UK

**Damith Rodrigo**

Sri Lanka

**Udo Rolle**

Germany

**Pol Maria Rommens**

Germany

**Avi Rosenberg**

USA

**Andrew Rosenstengel**

Australia

**Hai-Bin Ruan**

USA

**Vidyendaran Rudhran**

India

**Erin Rudzinski**

USA

**Wadia Rustom**

India

**John Rutka**

Canada

**Aly Saber**

Egypt

**Amitabh Saha**

India

**Edith Said**

Malta

**Mehmet Coskun Salman**

Turkey

**Carmelo Salpietro**

Italy

**Panagiotis Samaras**

Switzerland

**Dayananda Samarawickrama**

UK

**Athanasios Saratzis**

UK

**Aqiba Sarfraz**

Pakistan

**Maria Sarigianni**

Greece

**Takeshi Sasaki**

USA

**Kanji Sasaki**

Japan

**Terence Savaridas**

UK

**Sandro Sbordone**

Italy

**Marius Scarlat**

France

**Bernhard Schaller**

France

**Ami Schattner**

Israel

**Sam Schulman**

Canada

**Cara Sedney**

USA

**Joern Seeger**

Germany

**Stefan Seewald**

USA

**Salih Selek**

Turkey

**Sixten Selleng**

Germany

**Dhivakar Selvaraj**

India

**Seyedmojtaba Seyedmousavi**

Netherlands

**Daniel Shaerf**

UK

**Hetalkumar Shah**

India

**Abdul-Rahman Shahein**

Canada

**Mahesh Shanmugam**

India

**Sapna Sharan**

Canada

**Aanchal Sharma**

India

**Shilpa Sharma**

India

**Rohit Sharma**

India

**Sameh Shehata**

Egypt

**Sherif Shehata**

Egypt

**Manjunath Shenoy**

India

**Takahiko Shibahara**

Japan

**Satoru Shikata**

Japan

**Samuel Shinjo**

Brazil

**Manabu Shirotani**

Japan

**Jun Shoji**

USA

**Rachel Shparberg**

Australia

**Brian Shuch**

USA

**Aseem Shukla**

USA

**Dhaval Shukla**

India

**Bobby Siddiqui**

UK

**Christos Sidiropoulos**

USA

**Alicja Sieminska**

Poland

**Francesco Signorelli**

France

**Anjana Silva**

Sri Lanka

**Francisco Javier Silvestre**

Spain

**Charles Simone**

USA

**Jory Simpson**

Canada

**Chanchal Singh**

India

**T. Shantikumar Singh**

India

**Nicholas Slater**

Netherlands

**Víctor Slavutsky**

Spain

**Benjamin Smallheer**

USA

**Stuart Smith**

UK

**Henry Smithson**

Ireland

**Ioannis Sokolakis**

Greece

**Doriette Soler**

Malta

**Sang Heon Song**

South Korea

**Lei Song**

China

**Guy Soo Hoo**

USA

**Shashank Sood**

India

**Toma Spiriev**

Bulgaria

**Giuseppe Spoto**

Italy

**Thambipillai Sri Paran**

Ireland

**Samer Srour**

USA

**Zdenek Stach**

Czech Republic

**Joerg Stein**

Austria

**Paschalis Steiropoulos**

Greece

**Anna Stepniewska**

Italy

**Hugo St-Hilaire**

USA

**Jacob Strahilevitz**

Israel

**Yoshiyuki Suehara**

Japan

**Koichiro Sugimura**

Japan

**Ruifang Sui**

China

**Sumadiono**

Indonesia

**Tucheng Sun**

China

**Rafal Surmacz**

Poland

**Alexey Surov**

Germany

**Maarten Taal**

UK

**Mariam Tajir**

Morocco

**Zennure Takci**

Turkey

**Tsuneo Takebayashi**

Japan

**Robert Takes**

Netherlands

**Nagio Takigawa**

Japan

**Anil Takvani**

India

**Winston Tan**

USA

**Tadashi Tanaka**

Japan

**Nil Tekin**

Turkey

**Nina Tello-Winniczuk**

Mexico

**H. Thomas Temple**

USA

**Abdulkadir Tepeler**

Turkey

**Hardik Thakker**

India

**Jagdeep Thakur**

India

**Rokshana Thanadar**

USA

**Loukas Thanos**

Greece

**Calvin Thigpen**

USA

**C.R. Thomas**

USA

**Dick Tibboel**

Netherlands

**Holger Till**

Austria

**Elwina Timehin**

UK

**Hiroyuki Tokue**

Japan

**Dario Tomasini**

Italy

**Benedetta Tomberli**

Italy

**Vincenzo Tombolini**

Italy

**Christopher Tormey**

USA

**Konstantinos Toulis**

Greece

**D.E. Tran**

Canada

**Christine Tranchant**

France

**Jonel Trebicka**

Germany

**Gianluca Trifiro**

Italy

**Chen-Chi Tsai**

Taiwan

**Stella Tsai**

Taiwan

**Iraklis Tsangaris**

Greece

**Ioannis Tsarouhas**

Greece

**Dimitrios Tsetis**

Greece

**Parmenion Tsitsopoulos**

Sweden

**Norihiko Tsuchiya**

Japan

**Mehmet Turgut**

Turkey

**Stefano Uccella**

Italy

**Kenzo Uchida**

Japan

**Akin Ugras**

Turkey

**Burak Veli Ulger**

Turkey

**Claudio Ungari**

Italy

**Gabor Ungvari**

Australia

**Agahan Unlu**

Turkey

**Rashmi Upadhyay**

India

**Ofir Uri**

Israel

**Nicolas Useche**

Colombia

**Jiri Valenta**

Czech Republic

**Virginia Van Duyne**

USA

**Vincent Vander Poorten**

Belgium

**Ivor Vanhegan**

UK

**Prasad Vannemreddy**

India

**Nikolaos Vassos**

Germany

**P. Veena**

India

**Giovanni Vennarecci**

Italy

**Jose Ventura**

USA

**Stefano Veraldi**

Italy

**David Verity**

UK

**Paolo Verze**

USA

**Nereo Vettoretto**

Italy

**Kenneth Vick**

USA

**Goran Videnovic**

Serbia

**Ana Paula Vivas**

Brazil

**Theodosia Vogiatzaki**

Greece

**Jesse Waggoner**

USA

**Santosh Waigankar**

India

**Heather Wakelee**

USA

**Daniel Walsh**

UK

**Michael Walton**

UK

**Wei-Jie Wang**

Taiwan

**Hong Wang**

China

**Yuhui Wang**

China

**Alison Ward**

Canada

**David Warrell**

UK

**Atsuya Watanabe**

Japan

**Sama Weerapperuma**

Sri Lanka

**Öghdf Wefwfr**

Turkey

**Eva Wegner**

Australia

**Joachim Weis**

Germany

**Mandika Wijeyaratne**

Sri Lanka

**James Wilson**

USA

**James Winearls**

Australia

**Guido Woeste**

Germany

**Ronit Wollstein**

USA

**Samuel Ys Wong**

Hong Kong

**Seung Hoon Woo**

South Korea

**Sandy Wright**

Canada

**Tsung-Tien Wu**

Taiwan

**Shoji Yabuki**

Japan

**Naveen Yadav**

India

**Yoshitaka Yamaguchi**

Japan

**Akira Yamasaki**

Japan

**Hiroshi Yamazaki**

Japan

**Yoko Yanagisawa**

UK

**Yukihiro Yano**

Japan

**Alpaslan Yavuz**

Turkey

**Dali Yin**

USA

**Hiroshi Yoshida**

Japan

**Cheol Woong Yu**

South Korea

**Canan Yücesan**

Turkey

**Hideo Yuki**

Japan

**Zacharias Zachariah**

Switzerland

**Yehuda Zadik**

Israel

**Ahmed M. Zaki**

Egypt

**Apostolos Zatagias**

Greece

**Nick Zavras**

Greece

**Itai Zeevi**

Israel

**Philippe Zerbib**

France

**Haitao Zhao**

China

**Chunlong Zhong**

China

**Jun Zhong**

China

**Jasna Zidverc-Trajkovic**

Serbia

**Esther Zipperer**

Germany

**Richard Zoraster**

USA

**Konstantinos Zorbas**

Greece

**Giovanna Zorzi**

Italy

**Vasiliki Zouvelou**

Greece

**Alauldeen Zubair**

Iraq

**Ali Ersin Zumrutbas**

Turkey

